# Recognition and management of abdominal compartment syndrome among German anesthetists and surgeons: a national survey

**DOI:** 10.1186/2110-5820-2-S1-S7

**Published:** 2012-07-05

**Authors:** Torsten Kaussen, Jens Otto, Gerd Steinau, Jörg Höer, Pramod Kadaba Srinivasan, Alexander Schachtrupp

**Affiliations:** 1 Department of Pediatric Cardiology and Intensive Care, University Children's Hospital, Hannover Medical School (MHH), OE 6730, Carl-Neuberg St. 1, 30625 Hannover, Germany; 2 Department of Surgery, RWTH Aachen University Hospital, Pauwelsstrasse 30, 52074 Aachen, Germany; 3 Department of Surgery, Hochtaunus-Kliniken Bad Homburg, Urseler Str. 33, 61348- Bad Homburg v. d. Höhe, Germany; 4 Institute of Experimental Animal Science, RWTH Aachen University Hospital, Pauwelsstrasse 30, 52070 Aachen, Germany

**Keywords:** abdominal compartment syndrome, intra-abdominal pressure, intra-abdominal hypertension, intensive care unit, survey, questionnaire, bladder pressure, intra-vesical pressure measurement.

## Abstract

**Background:**

Abdominal compartment syndrome (ACS) is a life threatening condition that may affect any critically ill patient. Little is known about the recognition and management of ACS in Germany.

**Methods:**

A questionnaire was mailed to departments of surgery and anesthesia from German hospitals with more than 450 beds.

**Results:**

Replies (113) were received from 222 eligible hospitals (51%). Most respondents (95%) indicated that ACS plays a role in their clinical practice. Intra-abdominal pressure (IAP) is not measured at all by 26%, while it is routinely done by 30%. IAP is mostly (94%) assessed via the intra-vesical route. Of the respondents, 41% only measure IAP in patients expected to develop ACS; 64% states that a simpler, more standardized application of IAP measurement would lead to increased use in daily clinical practice.

**Conclusions:**

German anesthesiologists and surgeons are familiar with ACS. However, approximately one fourth never measures IAP, and there is considerable uncertainty regarding which patients are at risk as well as how often IAP should be measured in them.

## Introduction

Abdominal compartment syndrome (ACS) is defined as a persistent intra-abdominal pressure (IAP) of more than 20 mmHg accompanied by new organ dysfunction or failure. Left untreated, this condition has a high mortality rate [1-6]. Intra-abdominal hypertension (IAH) is defined by a sustained or repeated pathological elevation of IAP to more than 12 mmHg and is considered a precursor of ACS [1]. Both IAH and ACS may occur in any patient population requiring intensive care [7,8].

According to surveys in Canada, Great Britain, Australasia, Belgium, China and the USA, detection and management of IAH and ACS are inconsistent [2,4,9-16]. Familiarity with the devastating consequences of increased IAP is abundant; however, the relevance of ACS in routine care varies. There is no agreement regarding the indication for IAP measurement and its timing [4]. Moreover, the threshold for decompression is still a matter of debate, as prospective randomized trials are missing [1,10].

Whether a similar level of uncertainty concerning the recognition and management of ACS exists in Germany, and whether this may be related to the techniques available in clinical routine is unknown. We also speculate that a simple, more standardized technique might help improve monitoring of IAP. As comparable studies have yet to be published, we performed this one using a questionnaire.

## Methods

In 2006, a questionnaire (see Additional file 1) was sent to the head physicians of departments of surgery and anesthesia in hospitals with more than 450 beds in Germany. This 450-bed threshold was chosen because hospitals of this size are frequently teaching hospitals and serve as referral centers for smaller hospitals with elective or out-patient surgery.

According to these criteria, the hospitals were selected via an internet-based hospital registry http://www.krankenhaus.net. A total of 222 questionnaires were sent out. Recipients were asked to reply by fax within 2.5 months. No reminder was sent.

Statistical analysis was calculated using Statistical Package for Social Sciences 12.0.1 for Windows (SPSS Inc., Chicago, IL, USA). Some questions could have more than one answer; in these cases, results were analyzed for multiple responses. The answers were analyzed with respect to training completed by unpaired non-parametric testing (Mann-Whitney U).

## Results

A total of 113 questionnaires were returned, four were incomplete or unreadable. Excluding these, 109 questionnaires were analyzed (49%). Participants stated they had completed training either in anesthesiology (49%) or surgery (51%). Their indicated years of clinical practice averaged 21.8 (range 7 to 40).

**Figure 1:**
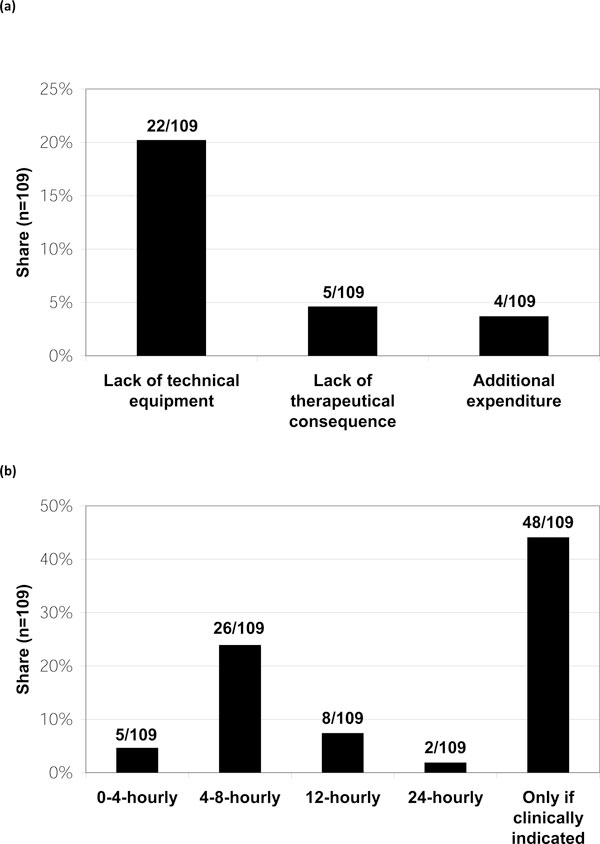
**Reasons for not measuring IAP and frequency of IAP measurements**. (**a**) Stated reasons for not measuring IAP. Out of 109 respondents, 28 denied regularly measuring IAP due to the reasons presented (% of respondents, multiple answers; question 2). (**b**) Frequency of IAP measurements among those who stated to measure IAP. Of the 109 respondents, 81 elaborated on when to measure IAP (% of respondents, multiple answers; question 2).

**Figure 2:**
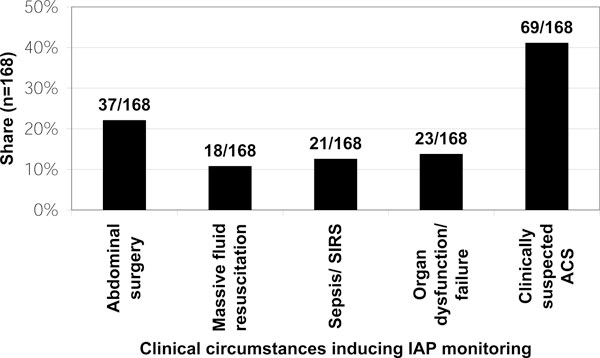
**Patient groups which are regularly IAP monitored**. Eighty-one stated their criteria regarding in which kind of patients IAP should be measured (% of respondents, multiple answers; question 4).

The majority (65%) stated ACS rarely plays a role in their clinical practice; 24% are concerned regularly; 6% often. Not more than 5% do not encounter this complication. Responding to 'Do you measure IAP?', 28 (26%) stated 'no'. Of those 81 respondents (73%) who measured IAP, 48 (59%) do so 'Only when clinically indicated'. Failure to establish an IAP measurement technique, cited by 22 respondents (28%, see Figure 1a, b), was the most common reason for not measuring it. The method indicated as most often used for IAP assessment was the measurement of intra-vesical pressure (bladder pressure measurement; 94%, multiple answers possible). In the other cases, a trans-gastric technique was reported. Multiple answers were possible for the question 'In which patients do you measure IAP?'. Respondents most often (41%) answered that measurement is only performed in patients thought likely to develop ACS (Figure 2).

**Figure 3:**
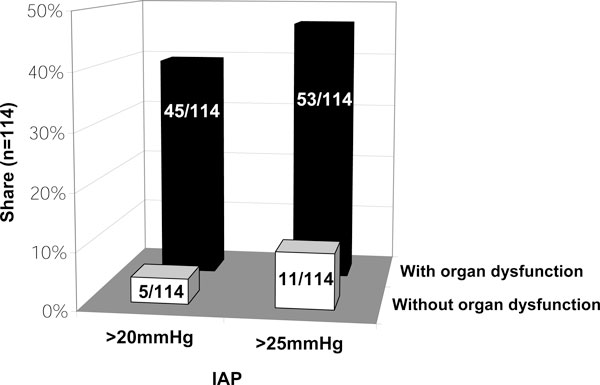
**Critical IAH threshold calling for surgical decompression dependent on organ function and dysfunction**. Ninety-four respondents stated their criteria concerning when performing decompressive laparotomy dependend on IAP and organ dysfunction (% of respondents, multiple answers; question 5)

The majority (86%) of respondents stated that the decision to surgically decompress is rather a matter of beginning organ dysfunction than of exceeding pressure thresholds (Figure 3). A simpler, more standardized application would lead to an increased use in 70 of 104 respondents (67%). Of the 26 participants not measuring IAP, even 77% think a simplified technique would improve acceptance.

## Discussion

Consensus definitions concerning ACS have been published in order to provide a basis for current treatment [1,3,8]. Prospective randomized trials are missing which is probably due to the variable incidence (1% to 15%), rapid progression and the disease pattern [17-19]. This situation leaves some questions open. Furthermore, the overall purpose of this survey was to study the current status in Germany.

### Awareness of ACS and performance of IAP measurements

According to our results, ACS plays a role in 95% of participants' clinical practice. About one third encounter ACS regularly or often. This is comparable to other countries where familiarity with ACS reportedly ranges from 73% to 99% of respondents (Table 1). More than one third of respondents from all over the world diagnose at least five cases of ACS each year. Although knowledge regarding ACS seems abundant, about one fourth of respondents claim they never measure IAP. In other surveys, the non-measuring rate was mostly comparable (range 2% to 80%; Table 1). How those participants (who do not measure IAP) establish the diagnosis of ACS remains unclear. Clinical examination of the abdomen has a sensitivity of only 50% to 60% which is similar to a coin toss [20-22]. Malbrain et al. demonstrated that also the abdominal perimeter is an inaccurate way for assessing increasing IAP [23].

**Table 1:** Comparison between results of current surveys related to IAH and ACS

Authors	Reference	Awareness of ACS	Yearly frequency of AS at ICUs	Performance of IAP measurements	Basis of IAH/ACS diagnosis	Measure method	Frequency of measurements	Threshold IAH	Threshold ACS	Experience with/opinion about DL and OA
Mayberry et al.	[9]	85%	14%: No cases	69% to 95%	66% IAP measure	IVP	59% If suspected	15 mmHg (11%)		86%: DL if IAH + OD (= ACS)
			52%: One to five cases		34% Clinical		6% Regularly	18 mmHg (22%)		14%: DL if IAH alone
			33%: Five cases					22 mmHg (31%)		If OA: Bag > absorb. Mesh > non-absorb. Mesh
								25 mmHg (12%)		
Kirkpatrick et al.	[10]	100%		52%	43% IAP measure	97% IVP			25 mmHg + OD	8%: DL if IAH alone
						3% IGP			34 mmHg - OD	90% OA after trauma surgery
										If OA: Bag > VAC > non-absorb. > absorb. Mesh
Ravishankar and Hunter	[4]	99%		76%	76% IAP measure	IVP	93% If suspected		20 mmHg (29%)	2%: DL if IAP > 20 mmHg alone (= IAH III)
					24% Clinical		4% After EL		25 mmHg (71%)	27%: DL if IAP > 20 mmHg + OD (= ACS)
							3% After EL + HVR			7%: DL if IAP > 25 mmHg alone (= IAH IV)
							15%: Zero to four hourly			64%: DL if IAP > 25 mmHg + OD (= ACS)
							27%: Four to eight hourly			
							11%: 12 hourly			
							3%: 24 hourly			
Nagappan et al.	[14]	92%	'Depending on used thresholds'; ICU-dependent	48% to 93%	8% Clinical	89% IVP	8% Never	12 mmHg (11%)	IAH + OD (69%)	92%: ACS = decompression (ever)
						39% Direct	53% Rarely	20 mmHg (64%)	≥30 mmHg - OD (33%)	64%: 'ACS should be treated regardless of IAH'
						6% IGP	19% Regularly			
						6% IRP	25% Often			
Tiwari et al.	[2]	73% to 97%			74% to 94% IAP measure	90% to 96% IVP			11 to 30 mmHg (teaching hospit.)	42% Performed DL in 0% to 25% of ACS patients
					60% to 77% Clinical	4% to 10% Direct			11 to 50 mmHg (district hospital)	19% Performed DL in 25% to 50% of ACS patients
					3% to 12% CT scan					16% Performed DL in 50% to 75% of ACS patients
					3% pH manometry					23% Performed DL in 75% to 100% of ACS patients
Kimball et al.	[15]	75% to 98%	17%: No cases	76% to 98%	70% IAP + clinical	IVP	47% Seldom	'Patient dependent'	20 to 27 mmHg (42%)	'Useful invasive therapy options':
			39%: One to three cases		20% Clinical		23% Often		12 to 19 mmHg (18% to 25%)	-Decompressive laparotomy
			27%: Four to seven cases		7% IAP measure		8% Routinely		12 to 19 mmHg (18% to 25%)	-Paracentesis/drains
			10%: Eight to 10 cases		3% Others		1% Other			-Escharatomy/fasciotomy
			8%: > Ten cases							-Peritoneal dialysis (catheter)
De Laet et al.	[12]	80%		41%	51% IAP measure	'Majority' IVP	59% Never	15 mmHg (IQR 12 to 15)	20 mmHg (IQR 20 to 20)	75% Performed at least one DL
					49% Clinical		28% If suspected			60% Performed at least one OA
							12% Continuously			If OA: Bag > abs. > VAC > gauze > non-absorb.
Ejike et al.	[13]			76%	76% IAP measure	68% IVP	27% Never			
					24% Clinical	13% Direct				
						+/- Doppler				
						+/- IGP				
Zhou et al.	[16]		0%: No cases	69%	31% Clinical	100% IVP	88% If suspected		25 mmHg	68%: First-line therapy paracentesis
			44%: One to three cases			7% CVP	71% Seldom			56%: DL if IAP > 25 mmHg + OD (= ACS)
			16%: Four to seven cases				29% Regularly			
			8%: Eight to ten cases				8% After EL			
			32%: > Ten cases				4% After HVR			
Kaussen et al^a^		95%	6%: Never	75%	26% Clinical	94% IVP	40% If suspected		20 mmHg (43%)	4%: DL if IAP > 20 mmHg alone (= IAH III)
			64%: Seldom			6% IGP	4%: Zero to four hourly		25 mmHg (57%)	39%: DL if IAP > 20 mmHg + OD (= ACS)
			24%: Regularly				22%: Four to eight hourly			10%: DL if IAP > 25 mmHg alone (= IAH IV)
			6%: Often				7%: 12 hourly			46%: DL if IAP > 25 mmHg + OD (= ACS)
							2%: 24 hourly			
Malbrain et al.	[11]	99%	0.3%: No cases	86%	69% IAP + clinical	92% IVP	42% If suspected	5 mmHg (< 1%)	20 mmHg (27%)	74%: DL if IAH + OD
							4% Continuously			
			62%: One to five cases		24% IAP measure	4% Direct	32% Four hourly	10 mmHg (6%)	25 mmHg (12%)	9%: DL if severe OD (even without IAH)
			20%: Six to ten cases		13% CT scan	3% IGP	26% Six to eight hourly	12 mmHg (18%)	> 25 mmHg (58%)	6%: DL dependent on cause of ACS
			6%: 11 to 15 Cases		10% Abdom. perimeter		6% 12 hourly	15 mmHg (25%)		If OA: VAC (39%) > Bag (24%) > mesh (21%)
			5%: 16 to 20 cases		8% Abdom. ultrasound		2% 24 hourly	20 mmHg (29%)		
			6%: > 25 Cases					25 mmHg (5%)		
								> 25 mHg (15%)		
								Others (2%)		
Newcombe et al.	[38]	88%		92%	83% IAP measure	93% IVP	21% Regularly		≤15 mmHg (11%)	
					8% IAP + clinical	7% Direct	54% Sometimes		≤25 mmHg (59%)	
					7% Clinical	0% IGP	19% Never		> 25 mmHg (30%)	

absorb., absorbable (mesh); abdom., abdominal; ACS, abdominal compartment syndrome; AustAsia, Australia and Asia (Australasia); Bag, 'bowel bag' such as 'Bogota bag'; CVP, central venous pressure measurement; direct, intra-abdominal pressure measurement via intra-abdominal placed probes; DL, decompressive laparotomy; EL, emergeny laparotomy; hospit., hospital; HVR, high-volume resuscitation; IAH, intra-abdominal hypertension; IAP, intra-abdominal pressure; ICU, intensive care unit; IGP, intra-gastric pressure measurement; IQR, inter-quartile range; IRP, intra-rectal pressure measurement; IVP, intra-vesical (bladder) pressure measurement; non-absorb., non-absorbable (mesh); OA, open abdomen management; OD, organ dysfunction/failure; VAC, vacuum-assisted. ^a^Unpublished work.

Among participants measuring IAP, the majority (59%) stated they perform measurements only if clinically indicated; in contrast, 30% advocate a routine measurement one to six times per day (Figure 1b). This appears to correlate with respondents tending to perform measurements mostly in patients *expected *to develop ACS (40%).

### IAP measurement methods

In accordance with all formerly published surveys, IAP measurement via the bladder is the most frequently used technique also in Germany (Table 1). Of the respondents, 70% stated that a simpler, more standardized technique would be used more often to assess IAP. This impression is supported by the finding that some respondents refuse bladder pressure measurement because the technique may 'not be established' or appears 'too complex in technical regards'. Both points of criticism appear unjustified. Several studies in humans as well as in animals proved replicability and reliability of the method [24,25]. Further, the measurement techniques have become increasingly simple and user-friendly over the last years, making it no longer possible to speak of an overly complicated IAP measurement technique. For example, the manometer technique, published by Harrahill in 1998 [26] and perfected by Lee [27], offers a maximum simplification of the bladder pressure test and requires no additional instruments other than a ruler and trans-urethral catheter. Using this principle, even commercially available measurement systems have been developed (for example Foleymanometer, Holtech^® ^medical, Charlottenlund, Denmark). Nevertheless, a minimum amount of training for personnel is required to avoid certain pitfalls. This includes, for example, ruling out a neurogenic or organic bladder dysfunction, ensuring sufficient relaxation of the local abdominal muscles, and the correct steady positioning of the patient with a continuous transparent reference point for the measurement of pressure equivalents.

Other indirect methods such as intra-gastric and intra-rectal pressure measurements rather constitute an exception than the rule and were stated to be performed by no more than 6% of respondents (Table 1). This is noteworthy in so far as different commercially available measurement systems, meanwhile, have been developed which allow to continuously monitor IAP levels via the stomach (for example CiMON^®^, Pulsion^® ^Medical Systems, Munich, Germany or 'IAP catheter', Spiegelberg^®^, Hamburg, Germany). Continuous measurement systems are able to minimize health care providers' workload as well as ensure non-stop observation of especially at risk patients. Pressure transducers, which are directly inserted into the abdomen, even more precisely reflect the IAP. Further information with respect to direct and indirect IAP measurement methods, as well as to continuous and intermittent techniques can be found on excellent reviews which have been published by Malbrain [28] and De Keulenaer [29].

If various measurement procedures are available, the illness and the dynamic of possibly increasing abdominal pressure should be considered. The higher the IAP, and respectively, the more quickly it is increasing, the sooner continuous pressure monitoring should be considered in order to begin the necessary therapeutic procedures, including invasive ones, in time. Apparently, it is of utmost importance that IAP be quantified when, as recommended by the World Society on the Abdominal Compartment Syndrome (WSACS), certain risk factors are present (Figure 2; Table 2). Using appropriate therapy algorithms, it should thereby become possible to react earlier and assertively enough to IAH that an ACS case does not even arise.

**Table 2:** Risk factors for IAH/ACS as proposed by the WSACS (adapted from [24])

Category	Risk factors
1. Diminished abdominal wall compliance	Mechanical ventilation, especially fighting with the ventilator and use of accessory respiratory muscles
	Use of positive end expiratory pressure (PEEP) or the presence of auto-PEEP
	Basal pleuropneumonia
	High body mass index
	Pneumoperitoneum
	Abdominal (vascular) surgery, especially with tight abdominal closures
	Pneumatic anti-shock garments
	Prone and other body positioning
	Abdominal wall bleeding or rectus sheath hematomas
	Correction of large hernias, gastroschisis or omphalocele
	Burns with abdominal eschars
	
2. Increased intra-luminal contents	Gastroparesis/gastric distension/ileus/colonic pseudo-obstruction
	Abdominal tumor
	Retroperitoneal/abdominal wall hematoma
	
3. Increased intra-abdominal contents	Liver dysfunction with ascites
	Abdominal infection (pancreatitis, peritonitis, abscess, etc.)
	Hemoperitoneum/pneumoperitoneum
	Acidosis (pH below 7.2)
	
4. Capillary leak	Hypothermia (core temperature below 33°C)
	Polytransfusion/trauma (> 10 units of packed red cells/24 h
	Coagulopathy (platelet count below 5,000/mm^3^, an activated partial thromboplastin time (aPTT) more than 2 times normal, a prothrombin time (PTT) below 50%, or an international standardized ration (INR) more than 1.5)
	Sepsis (as defined by the American-European Consensus Conference definitions)
	Bacteremia
	Massive fluid resuscitation (> 5 l of colloid or crystalloid/24 h with capillary leak and positive fluid balance)
	Major burns

### IAP thresholds

Although the WSACS published definitions more than 5 years ago [3], there is still a remarkable lack of knowledge concerning the recommended threshold values in relation to IAH and ACS (Table 1). On the one hand, this might be caused by a lack of awareness of current literature; on the other, this might be influenced by personal experience, which might differ from published results and consensus. While the values gathered in the course of the surveys were partially over the WSACS limits for adults, the majority of pediatricians reported much lower values. This reflects the clinical impression that IAH and ACS can appear at much lower levels of abdominal pressure in children. In the framework of the 5th WSACS World Congress 2011 and using the data available at that time, Ejike et al. correctly demanded the establishment of pediatric limits (IAH: IAP > 10 mmHg, ACS: IAH + new organ dysfunction) (KT et al., unpublished work) [30].

### Surgical therapy options

Most of our respondents decide to decompress the abdomen based on the presence of organ dysfunction or failure in combination with IAH (Figure 3). The attitude towards the critical threshold (> 20 mmHg or > 25 mmHg) divides respondents into two groups of similar size (39% vs 46%). This is comparable to the surveys done by Ravishankar and Mayberry ([4,9], Table 1). One reason may be the lack of evidence as prospective outcome studies are missing and the mortality rate of ACS has remained high despite decompression [18,31]. Tiwari describes a reluctance among surgeons to operate patients with ACS [2]. They probably try to avoid complications associated with decompression and the management of an open abdomen as described by Kirkpatrick et al. in their survey of Canadian surgeons [10]. This restraint might arise from reports about sudden deaths following surgical decompression in patients suffering from IAH and ACS [32-34]. Fatal outcome in these patients might be related to fatal pulmonary embolism caused by venous stasis in the splanchnic venous capacitance pool during IAH/ACS. It has also been stated that lethal acute circulatory collapses and asystolia after decompression might be caused by the release of anaerobic metabolic products and inflammatory mediators from prior less perfused tissues (ischemia-reperfusion syndrome [35,36]). This pathogenesis, however, is not generally accepted.

Cheatham and Safcsak have demonstrated that routinely monitoring adult patients at risk and a stage-by-stage-guided therapy algorithm comprising medical as well as surgical options may considerably reduce patient mortality by up to 50% [37]. This also supports not delaying decompression when necessary. Respondents as well as participants in other surveys are familiar with decompressive laparotomy and more or less perform this escalated therapy option partly in combination with open-abdomen management often (Table 1). In this connection, it should be noted that, in all studies, the majority of physicians interviewed work in tertiary care hospitals and high-level ICUs. To a lesser degree, these results reflect circumstances found in basic and regular care hospitals where recognition and standardized therapy of IAH and ACS seem to lead a miserable existence.

### Limitations

Surveys are known to have limitations as results represent personal assessment rather than objective data. A limitation might be that the survey was only sent to the heads of departments and not to section members. It can be argued that the majority of head physicians carry out more administrative than clinical-curative tasks; meaning, they may not be sufficiently informed about current developments in the treatment of IAH and ACS which could have had a negative impact on the validity of the survey results. On the other hand, it appears less likely that establishment of IAP measurements nor therapeutic procedures, including decompressive laparotomies, are carried out in a department without the decision of the head of the department to do so. As a result, head physicians, even if less involved in everyday clinical work, are considered to be sufficiently knowledgeable to answer the questions posed.

A further limitation is that participants might have simply used their gut feeling instead of clinical databanks to answer the questions. Since doing so would cause more work, it must be assumed that the response rate would have been worse (range of response rates of published IAP surveys: 6% to 90%; Table 3). Therefore, it was decided not to perform a databank survey. The results, which are, to a great extent, identical to the available literature, appear not to express an undue bias (Table 1).

**Table 3:** Overview and structural description of current surveys related to IAH and ACS

Authors	Reference	Country	**Year**^a^	Questionnaires (returned/sent)	Response rate	Communication channel	Specialty of participitants	Level of medical care
Mayberry et al.	[9]	USA	1999/1997	292/473	62%	Mail	Trauma surgeons	85% Teaching hospitals
Kirkpatrick et al.	[10]	Canada	2005/2005	86/102	84%	Mail and email	Trauma surgeons	
Ravishankar and Hunter	[4]	UK	2005/NA	137/207	66%	Mail	Intensivists	
Nagappan et al.	[14]	Australasia	2005/2004	36/40	90%	Hand-out at workshop	ICU registrars	72% High-level ICU
								10% Medium-level ICU
								3% Low-level ICU
Tiwari et al.	[2]	UK	2006/2004	127/222	57%	Mail	Intensivists	25% Teaching hospitals
								75% District hospitals
Kimball et al.	[15]	USA	2006/2001	1622/4538	36%	Mail	35% Surgeons	
							32% Internists	
							18% Pediatricians	
							10% Anesthetics	
							1% Emergency doctors	
De Laet et al.	[12]	Belgium	2007/2005	41/689	6%	Email	Surgeons	73% Teaching hospitals
								27% District hospitals
Ejike et al.	[13]	60% America	2010/2006	517/1107	47%	Hand-out at pediatric congresses	60% Pediatric nurses	81% Tertiary care hospitals
		26% Europe					30% Pediatric intensivists	14% Community hospitals
		12% Australasia					4% General pediatricians	2% Private practise
							6% Other pediatric health care providers	1% Clinics
								2% Others
								
Zhou et al.	[16]	China	2011/2010	108/141	77%	Mail	39% Emergency doctor	100% Tertiary care hospitals
							36% Internists	
							19% Surgeons	
							6% Anesthetics	
Kaussen et al.		Germany	2012^b^/2006	113/222	51%	Mail	52% Surgeons	Larger hospitals with > 450 patient beds
							48% Anesthetics	
Malbrain et al.	[11]	58% America	2012/2007	2244/8081	28%	Contacting via email/online-questionnaire	37% ICU physicians	
		32% Europe					23% Surgeons	
		9% Australasia					21% Anesthetics	
		1% Africa					8% Internists	
							6% Pediatricians	
							2% Emergency physicians	
							1% Cardiologists	
							2% Others	
Newcombe et al.	[38]	97% USA	2012/2010	433/691		Hand-out at pediatric congress	Pediatric nurses	> 60% Tertiary care hospitals
								< 30% Community hospitals
								< 10% Others

Australasia, Australia and Asia; ICU, intensive care unit. ^a^Contains 2 annual details: 1st, year of publication; 2nd, year of conduction of underlying study/survey. ^b^Unpublished work.

It was decided to send questionnaires to intensive care units of surgical and anesthesiological departments. Due to the current structure in Germany, patients with IAH/ACS are predominantly placed in departments of surgery and anesthesiology and by far less often present in internal departments.

However, the data display an attitude towards the management of ACS in Germany, thereby, demonstrating a lack of consensus and certainty. This might help guide future studies with a multi-center prospective randomized approach.

## Conclusion

ACS is known among German anesthesiologists and surgeons, and both groups do not differ in their attitude towards this complication. Measurement of bladder pressure appears to be the current standard to assess IAP. However, about one fourth of responding physicians in Germany never measure IAP, and there is considerable uncertainty about which patients are at risk of developing ACS and how often IAP should be measured. Regarding the IAP threshold for decompression (20 or 25 mmHg), respondents remain undecided. These findings lead to the overall impression that recognition and management of IAH or ACS need to be further established in Germany.

## Abbreviations

ACS: abdominal compartment syndrome; IAH: intra-abdominal hypertension; IAP: intra-abdominal pressure; WSACS: World Society on the Abdominal Compartment Syndrome.

## Competing interests

In addition to his assistant professorship at the Technical University of Aachen (Germany), Alexander Schachtrupp is head of the Department of Medical Sciences at B. Braun Melsungen in Germany. B. Braun does not distribute any medical devices or products concerning the diagnosis and/or treatment of IAH or ACS. The other authors declare that they have no competing interests.

## Authors' contributions

Literature research was done by JO, TK and AS. Data collection was mainly performed by JO and AS. The article was written by TK, JO and AS and reviewed by GS, JH and AS. PKS delivered linguistic advice and substantially revised the manuscript. All authors read and approved the final manuscript.

## Supplementary Material

Additional file 1 **Appendix**. Click here for file
