# Supplementary material for: Recognition and management of abdominal compartment syndrome among German anesthetists and surgeons: a national survey
Source: Ann Intensive Care. 2012 Jul 5;2(Suppl 1):S7. doi: 10.1186/2110-5820-2-S1-S7 (PMC3390300; doi:10.1186/2110-5820-2-S1-S7)
Supplement: Additional file 1 — Appendix. [file 2110-5820-2-S1-S7-S1.docx]

**Appendix**

1) Does the abdominal compartment (ACS) play a role in your clinical practice?

-No

-Yes, rarely

-Yes, regularly

-Yes, often

2) Do you measure the intra-abdominal pressure (IAP)?

No, because: -Technique is not established in our institution

-Results do not have a relevant influence on further therapy

-Technical complexity is too high

If yes, how often do you measure IAP?

-zero to four hourly

-four to eight hourly

-12 hourly

-Once every 24 h

-Only when clinically indicated

3) Which method do you most commonly use to measure IAP?

-Intra-vesical pressure measurement

-Intra-gastric pressure measurement

-Other (please specify)

4) In which patients do you measure IAP?

-In post-operative ICU patients following abdominal surgery

-Patients exposed to massive fluid resuscitation

-Patients with sepsis/systemic inflammatory response syndrome

-Patients with organ dysfunction/failure

-Only those patients thought likely to develop abdominal compartment syndrome

5) When would you recommend surgical decompression?

-IAP persistently > 25 mmHg irrespective of whether signs of organ dysfunction are present or not

-IAP persistently > 25 mmHg together with signs of organ dysfunction

-IAP persistently > 20 mmHg irrespective of whether signs of organ dysfunction are present or not

-IAP persistently > 20 mmHg together with signs of organ dysfunction

6) Would a simpler and standardized application (e.g., measurement of central venous pressure) lead to an increased use?

-Yes

-No

7) Field

Years of clinical practice

Completed training -Yes

-No
